# Genome Sequence of a Virulent African Swine Fever Virus Isolated in 2020 from a Domestic Pig in Northern Vietnam

**DOI:** 10.1128/MRA.00193-21

**Published:** 2021-05-13

**Authors:** Quang Lam Truong, Thi Lan Nguyen, Thi Hoa Nguyen, Jishu Shi, Hiep Lai Xuan Vu, Thi Lan Huong Lai, Van Giap Nguyen

**Affiliations:** a Key Laboratory of Veterinary Biotechnology, Faculty of Veterinary Medicine, Vietnam National University of Agriculture, Hanoi, Vietnam; b College of Veterinary Medicine, Kansas State University, Manhattan, Kansas, USA; c Nebraska Center for Virology, University of Nebraska—Lincoln, Lincoln, Nebraska, USA; DOE Joint Genome Institute

## Abstract

This study reports the genome sequence of an isolated African swine fever (ASF) virus (VNUA-ASFV-05L1/HaNam) obtained at the fourth passage on pulmonary alveolar macrophages. The virus was isolated during a typical acute ASF outbreak in pigs in a northern province of Vietnam in 2020.

## ANNOUNCEMENT

Spreading to nearly all major swine-producing countries, African swine fever (ASF) is currently considered one of the most important transboundary diseases of pigs (1). The etiological agent, ASF virus (ASFV), belongs to the *Asfarviridae* family, *Asfivirus* genus, and possesses an “open” pangenome (2, 3). Of the 24 ASFV genotypes (I to XXIV) known to date (4), only genotypes I and II have been detected outside Africa (5). The genomic complexity of ASFV is reflected by (i) a very large DNA genome (averaging 186,817 bp), (ii) a variable length from 170,101 bp to 193,886 bp, and (iii) the presence of hundreds of open reading frames, which are classified as core genes or accessory genes (2, 3). As a result, continuous genomic characterization of ASFV genomes is essential for diagnostic, epidemiological, and vaccine development purposes.

In this study, we isolated a virulent strain (VNUA-ASFV-05L1/HaNam) from the spleen of a fattening pig that had succumbed to an acute infection. The sample was taken from a small-scale farm of a farrow-to-finish production system located in a northern province of Vietnam (Ly Nhan District, Ha Nam Province). The spleen homogenate was filtered through a 0.45-μm membrane and inoculated in pulmonary alveolar macrophages (PAMs). The infected PAM culture showed specific cytopathic effect at 48 h postinoculation, as characterized by hemadsorption dose (HAD). At the fourth passage, the virus titer was 7.14 log_10_ 50% HAD (HAD_50_)/ml at 72 h postinfection. The virus was then semipurified and concentrated using an Amicon Ultra-15 centrifugal filter unit (UFC901024; Millipore). Total DNA was extracted with the QIAamp DNA minikit (51304; Qiagen). The extracted DNA was checked for integrity by gel electrophoresis and measured as at least 50 ng/μl.

The next-generation sequencing was conducted by Apical Scientific Sdn Bhd (Selangor, Malaysia). The library was constructed with the NEBNext Ultra DNA library preparation kit, and the sequencing platform was an Illumina NovaSeq 150PE system. Primer sequences were removed from raw Illumina reads using BBDuk of the BBTools package (https://jgi.doe.gov/data-and-tools/bbtools). Quality control reads were assembled *de novo* using SPAdes (6) and polished using Pilon v1.23 (7), implemented in Unicycler (8). All contigs were subjected to BLASTN searches against the NCBI nucleotide database. Open reading frames were predicted using Prodigal (9) and annotated using Prokka v1.14.6 (10). The single contig (VNUA-ASFV-05L1/HaNam) with a BLASTN similarity to an ASFV was aligned with a number of reference genomes using MAFFT v7.450 (11). The pairwise comparison of average nucleotide identity (ANI) between ASFV genomes was performed by ANI Calculator (12) (https://www.ezbiocloud.net/tools/ani). Other tools for genomic visualization and classification of multigene family (MGF) proteins in ASFV were geneCo (13) and MGFC (14), respectively. All bioinformatic tools were run with default parameter settings.

The total output of the sequencer was 10,353,104 reads, and the total number of reads that mapped to the reference genome (GenBank accession number FR682468) was 461,934. The reads were able to yield a longest single linear contig of 186,237 nucleotides, which was confirmed to be ASFV based on the BLAST result (query coverage, 100%; percent identity, 99.89% to 99.99%). The 186,237-nucleotide genome of VNUA-ASFV-05L1/HaNam had a mean GC content of 38.47% and was predicted to contain 158 protein-coding genes. Compared to several recently reported p72 genotype II strains, the genome-wide sequence identity of VNUA-ASFV-05L1/HaNam was 99.95% with respect to Georgia 2007/1 (FR682468) and 99.98% with respect to InnerMongolia-AES01 (MK940252). As a molecular marker distinguishing between closely related ASFVs, the tandem repeat sequence (TATATAGGAA) between the I73R and I329L genes of VNUA-ASFV-05L1/HaNam was classified as variant II (15). In terms of genomic organization, VNUA-ASFV-05L1/HaNam had all 86 core genes reported previously (3). VNUA-ASFV-05L1/HaNam contained all five MGFs (MGF-100, MGF-110, MGF-300, MGF-360, and MGF-505). The VNUA-ASFV-05L1/HaNam MGFs were characterized by a typical organization of (i) location at both the 5′ and 3′ ends and (ii) distribution along the plus and minus strands of the ASFV genome (14). Additionally, among 31 MGF proteins (14), MGF-110E, MGF-110H, and MGF-110I were not detected in the genome of VNUA-ASFV-05L1/HaNam.

### Data availability.

This genome sequence has been deposited in GenBank under the accession number MW465755. The raw reads are available in the SRA under the accession number SRX10287451.
